# Meta-analysis of cognitive, emotional, and behavioral predictors of academic achievement in higher education students

**DOI:** 10.3389/fpsyg.2026.1910328

**Published:** 2026-08-06

**Authors:** Feifei Xue, Minjian Wang, Lijuan Xi

**Affiliations:** Shenzhen Polytechnic University, Shenzhen, Guangdong, China

**Keywords:** academic achievement, cognitive predictors, emotional factors, higher education, self-regulated learning

## Abstract

**Background:**

Higher education outcomes are frequently associated with cognitive ability and the outcomes of previous school years, but there are likely emotional and behavioral factors that contribute to these as well. This meta-analysis examined the relative effects of cognitive, emotional, and behavioral predictors on academic performance in university and college students.

**Methods:**

Seventeen studies with higher education students were included. Eligible studies reported on at least one cognitive predictor (e.g., prior GPA, working memory, metacognition), emotional predictor (e.g., emotional intelligence, achievement emotions, emotion regulation), or behavioral/self-regulatory predictor (e.g., study habits, time management, engagement, procrastination) of one of the outcomes (GPA, course grades, or pass/fail status). Random effects models were used to transform effect sizes to Fisher’s z and pooled. Subgroup and meta regression analyses were used to investigate heterogeneity and moderators (design, field, region, measurement characteristics) while multiple diagnostics were used to assess publication bias.

**Results:**

The cognitive domain showed the strongest pooled association with academic achievement, r = 0.197, 95% CI [0.037, 0.347]. Emotional predictors showed a weak and imprecise association with academic achievement, r = −0.105, 95% CI [−0.376, 0.182]. Overall, the pooled effect across all predictors was small and not statistically strong, r = 0.017, 95% CI [−0.170, 0.202], with very high heterogeneity. Behavioral predictors showed an unstable estimate, r = 0.002, 95% CI [−0.477, 0.481], and should be interpreted cautiously.

**Conclusion:**

Taken together, the corrected synthesis suggests that cognitive predictors are the most consistent domain, whereas emotional and behavioral findings are less stable and require cautious interpretation. Cognitive predictors appear to be the most reliable domain in the current evidence base, while emotional and behavioral predictors remain tentative. Therefore, interventions that integrate cognitive assistance with specific training in SRL and emotion management are more in line with the evidence than interventions based on performance only.

## 1 Introduction

The academic outcomes of higher education are influenced by a wide range of personal factors that relate to academic performance beyond intellectual capacity such as emotional functioning and everyday behavior (Shengyao et al., 2024; Sultanova et al., 2024). The importance of working memory, metacognitive skills and self-regulation for engaging with complex learning tasks and for maintaining academic achievement has been emphasized in recent research. Research in affective and personality psychology suggests that emotional intelligence, subjective well-being and activity-based achievement emotions (e.g., enjoyment, boredom, frustration) are important correlates of achievement and persistence in the university context. Meanwhile, behaviors like showing up, studying, and working on academic tasks have been consistently tied to achievement, which means that what students do each day could be reflecting cognitive and emotional capacities into observable achievement (Pérez-González et al., 2022a; Zheng et al., 2023).

There have been a few meta-analyses that have combined subsets of this literature, but these tend to be narrow, targeting only one aspect of predictors. However, large-scale quantitative syntheses have concluded small-to-moderate, but significant, relations between subjective well-being and academic achievement, and strong relations between emotional intelligence and performance even after accounting for intelligence and personality factors (Davey et al., 2011; Sánchez-Álvarez et al., 2020). Other reviews indicate that activity-related emotions like enjoyment and boredom have been systematically correlated to academic results, and that engagement (cognitive, emotional, and behavioral) has moderate correlations with achievement at educational levels. Cognitive syntheses also highlight the role that executive functions and metacognitive strategies play in university students’ performance, especially in challenging learning settings. These are not, however, bodies of work that have examined the co-occurrence of cognitive, emotional and behavioral predictors and their common contribution to achievement in higher education populations (Pretorius and Heyns, 2026; Wu and Was, 2023).

This disintegration restricts theoretical and practical advances. Cognitive, emotional, and behavioral predictors are hard to compare across domains if analyzed in separate meta-analyses, and it remains unclear whether there is redundancy between predictors (e.g., metacognition vs. self-regulation) or whether any predictor adds a unique explanatory power when analyzing academic achievement in higher education (Koi, 2026; Xue and Khalid, 2026). Furthermore, many primary studies and reviews combine school and university students and may mask levels of development, context and autonomy that vary by school and university levels (Lee and Yang, 2022). Finally, there is evidence from low-resource higher education environments that psychosocial and cognitive resources could have different interactions when constrained in some way, but such evidence has yet to be synthesized quantitatively in a comprehensive, quantitative review.

This meta-analysis aims to provide a quantitative synthesis of the associations between cognitive (e.g., intelligence, working memory, metacognition), emotional (e.g., emotional intelligence, achievement emotions, subjective well-being), and behavioral (e.g., academic engagement and study behaviors) factors and academic achievement among higher education students using the most recent empirical evidence. Previous reviews have primarily examined individual constructs or specific theoretical perspectives; however, few have quantitatively compared these major domains within a unified meta-analytic framework. Accordingly, this study addresses three important gaps by: (a) estimating and directly comparing pooled effect sizes across cognitive, emotional, and behavioral domains; (b) synthesizing evidence across these domains within a single quantitative framework; and (c) examining potential contextual and methodological moderators, such as field of study, assessment type, geographical region, and economic context, where sufficient data were available. The primary hypothesis was that each domain would demonstrate a significant positive association with academic achievement. By clarifying the relative strength of these associations, this review contributes to a more comprehensive understanding of academic success in higher education and may help inform future educational strategies and subsequent intervention research.

## 2 Related work

In higher education, the focus of research on predictors of academic achievement has been on cognitive factors like prior grades and standardized test scores. Recent research has established that academic performance prior to entering university is one of the most robust individual predictors of university GPA and progression, but also indicates that this predictor varies across programs and domains of knowledge (Ackerman and Kanfer, 2025; Binder et al., 2019). The results of large-scale longitudinal studies suggest that early academic success varies greatly across students, with much of that variability being attributable to students’ prior grades and content-specific knowledge, and that learning strategies and motivation can account for this variation over time. These findings suggest that there is a need for continued cognitive preparation without which success in a higher education context would be less likely to occur (Steinmayr et al., 2019).

At the same time, an expanding literature highlights the role of psychosocial and non-cognitive factors in shaping student achievement. Studies that integrate cognitive abilities with psycho-social resources show that self-efficacy, perceived social support, and stress-coping skills add explanatory power over and above traditional academic indicators (Fong et al., 2017; Gutiérrez Tapia et al., 2019). Reviews of factors affecting student success emphasize that pre-entry metrics such as high school GPA must be considered alongside students’ ongoing use of university resources, quality of faculty interaction, and sense of belonging if institutions aim to predict and improve outcomes such as GPA and retention (Chongjin et al., 2025).

More recent work focuses specifically on emotional and social–emotional skills as predictors of academic outcomes in higher education. Empirical studies and narrative reviews show that emotional intelligence, emotion regulation, and socio-emotional skills are linked to persistence, engagement, and performance, often through their influence on study approaches and learning engagement (MacCann et al., 2020; Suson et al., 2025). For example, emotional learning interventions and broader socio-emotional competencies have been shown to improve students’ psychological resources and classroom behavior, which in turn support better academic outcomes. These results suggest that emotional functioning is not only an outcome of academic experiences but also a key driver of how students respond to academic demands (Suson et al., 2025).

Behavioral and engagement-related factors represent a third major strand of this literature. Research in higher education on engagement, study approaches and SRL behaviors indicates that factors like regular attendance, use of time, learning strategies, and active participation in learning activities are correlated with short-term learning performance as well as longer-term performance, even after controlling for prior achievement (David et al., 2024; Zhang and Kim, 2024). In recent studies on cognitive, emotional and behavioral involvement in university classes, it has been shown that both of these aspects of involvement have an impact on study approaches which in turn impacts on achievement (Castro-Lopez et al., 2025; Hasanov et al., 2021). Combined, current studies are moving towards a multi-dimensional perspective, with cognitive, emotional, and behavioral predictors working interdependently to predict academic achievement, which itself points to integrative models and quantitative syntheses that examine the relative and specific contributions of these predictors (Hasanov et al., 2021).

## 3 Methods

### 3.1 Study design and protocol

The study is a systematic review and meta-analysis on cognitive, emotional and behavioral predictors affecting academic achievement of higher education students. The review process was guided by the Preferred Reporting Items for Systematic Reviews and Meta Analyses (PRISMA 2020) guidelines for identification, screening, and eligibility assessment of included studies, and their reporting. The research question was determined *a priori* and eligibility criteria was determined within a conceptual framework that differentiated three predictor domains: cognitive (e.g., prior academic performance, cognitive ability, working memory); emotional (e.g., emotional intelligence, emotion regulation, resilience); and behavioral/self-regulatory (e.g., learning strategies, self-control, procrastination).

### 3.2 Search strategy

The database search was carried out from the inception of the databases to May 2026 in major bibliographic databases (Web of Science, Scopus, PsycINFO, PubMed, ERIC, Google Scholar). The search strategy used controlled vocabulary and free text terms that included the following categories: academic achievement terms (e.g., “academic achievement,” “course grade,” “GPA,” “grade point average,” “undergraduate”); higher education terms (e.g., “university students,” “college students,” “undergraduate”); and predictor constructs within the three domains (e.g., “cognitive ability,” “working memory,” “executive function,” “emotional intelligence,” “emotion regulation,” “self-regulation,” “self-control,” “study skills,” “learning strategies,” “procrastination”). Search strings were tailored for each database according to the syntax of that particular database. Study identification, screening, eligibility assessment, and inclusion were done according to the reporting guideline of PRISMA 2020. Each screening record was checked independently to confirm consistency between study selection results, quantitative data used for meta-analysis and the PRISMA flow diagram. Before statistical analyses, totals, reasons for exclusion, and final numbers of studies were crosschecked.

### 3.3 Eligibility criteria

Studies were eligible for inclusion if they met all of the following criteria: Participants were enrolled in higher education (universities, colleges, or equivalent post-secondary institutions). The study included at least one cognitive, emotional, or behavioral/self-regulatory predictor (e.g., prior academic performance, standardized test scores, cognitive tasks, emotional intelligence, emotion regulation, resilience, self-efficacy, self-control, learning strategies, study skills, procrastination). The primary outcome was academic achievement, operationalized as grade point average (GPA), course or exam grades, progression or success/failure, or comparable academic performance indicators. The study employed a quantitative empirical design (cross-sectional, longitudinal, or predictive modeling), conducted in naturalistic higher education settings. Sufficient information was provided to describe sample and design characteristics for qualitative synthesis.

For inclusion in the meta-analysis, studies additionally had to report at least one extractable effect size (e.g., Pearson’s correlation coefficient, odds ratio or adjusted odds ratio, standardized regression coefficient) or provide sufficient statistics from which such an effect size could be computed. Studies were excluded if they: (a) focused on non-tertiary populations (e.g., primary or secondary school students, non-student adults), (b) were intervention trials that did not report relevant baseline associations between predictors and achievement, (c) did not include an academic achievement outcome, (d) did not assess cognitive, emotional, or behavioral predictors, or (e) reported results only qualitatively or without sufficient statistical detail for effect-size extraction.

### 3.4 Study selection

**Figure 1:**
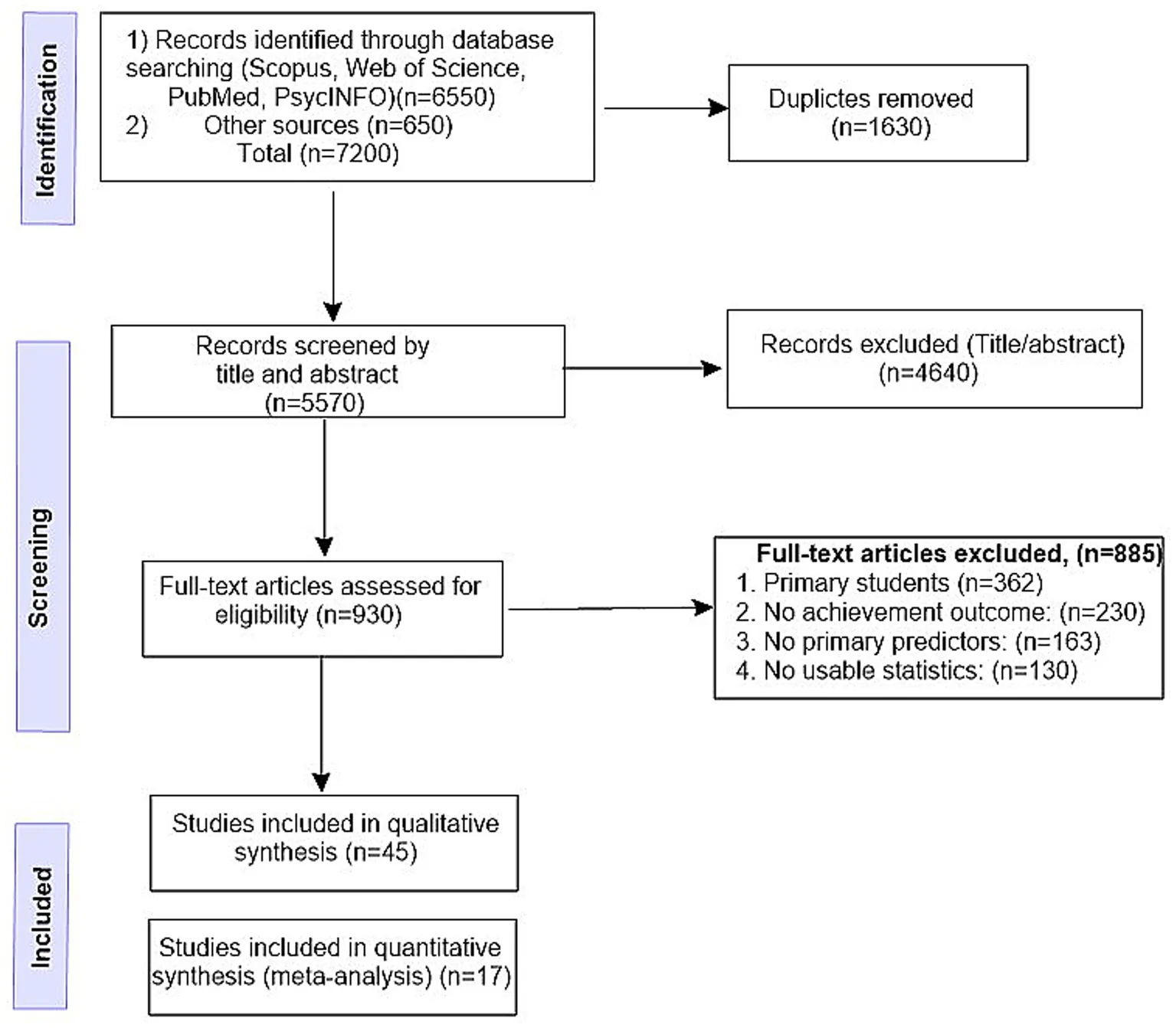
PRISMA 2020 flow diagram of study identification, screening, eligibility assessment, and inclusion (*k* = 45 studies).

All records retrieved from the database searches were exported into reference-management software, where 1,630 duplicate records were identified and removed. Following deduplication, 5,570 records underwent title and abstract screening, during which 4,640 records were excluded because they did not meet the predefined eligibility criteria. The remaining 930 articles were retrieved for full-text assessment. After detailed eligibility evaluation, 885 full-text articles were excluded for the following reasons: studies involving primary-school populations (*n* = 362), absence of an academic achievement outcome (*n* = 230), failure to examine the predefined cognitive, emotional, or behavioral predictors (*n* = 163), and insufficient statistical information for effect-size extraction (*n* = 130). Consequently, 45 studies were included in the qualitative synthesis, of which 17 studies provided extractable effect sizes and were included in the quantitative meta-analysis, as illustrated in the PRISMA 2020 flow diagram (Figure 1).

### 3.5 Data extraction

A standardized data extraction template was developed to ensure consistent coding across studies. For each eligible study, information on the author and year, country, study design, sample size, participant age, population or discipline, main predictor variables, categorized into cognitive, emotional, and behavioral domains, academic outcome measures, predictor-achievement relationships, and potential moderators or contextual factors was extracted for the qualitative synthesis (Table 1).

**Table 1:** Characteristics of included primary studies (45 studies).

Author (year)	Country	Study design	Sample size (n)	Age	Population	Main variables	Outcome measure	Key finding	Moderator(s)
Anmarkrud et al. (2019)	Norway	Review article	Not applicable	Not reported	Multimedia learning studies	Cognitive load, working memory	Learning outcomes (reviewed)	Weak measurement consistency reported	Not applicable
Barchard (2003)	United States	Quantitative hierarchical predictive design	117	Mean = 19.8 years (SD = 3.31)	Undergraduate psychology students	7 EI measures (ability & trait); controls: SAT & Big Five	Official year-end cumulative GPA	EI failed to predict unique variance in GPA (ΔR^2^ < 0.01) once SAT and Big Five were controlled	EI measurement model (ability vs. trait); control context (cognitive vs. personality)
Chew et al. (2013)	Malaysia	Cross-sectional	163 (84 first-year, 79 final-year)	Not stated in your snippet	Medical students	Emotional intelligence	Continuous assessment scores; final examination scores	Higher EI predicted better continuous assessment and final examination performance	Year of study (first vs. final year); gender; ethnicity
Dvorak (2024)	Sweden	Cross-sectional	107	25.83 ± 6.32	University teacher education students	Inhibitory control (Stroop task)	Grade average (GPA proxy)	Weak negative correlation found	Europe; developed; education field
Getahun Abera (2023)	Ethiopia	Cross-sectional	111	Not reported	University students	Emotional intelligence, pro-social behavior	Academic performance (GPA)	EI not significant; prosocial negative	Africa; LMIC; university students
Kassaw and Demareva (2024)	Ethiopia	Cross sectional	362	18–30	Undergraduate university students	Emotional coping, academic self-perception, social media use	Low academic achievement (GPA < 3.18)	Poor emotional coping, high social media use, and poor academic self-perception significantly predicted low academic achievement	NR
Kassaw and Demareva (2025)	Ethiopia	Cross-sectional	30	22.3 ± 4 years	Undergraduate health sciences students	Cognitive and psychosocial predictors	Grade point average (GPA)	Coping and memory predicted GPA	Africa; LMIC; health sciences
Kimo and Ayele (2022)	Ethiopia	Cross-sectional	360	Not reported	First-year university students	Social competency, coping strategies	Semester GPA	Social competency predicted GPA	Africa; LMIC; first-year students
Mekonen et al. (2017)	Ethiopia	Cross-sectional	592	22.16 ± 1.85	Medical students	Cognitive, behavioral, psychosocial predictors	Self-reported GPA	Substance use, age, entrance score significant	Africa; LMIC; medical students
Mohzan et al. (2013)	Malaysia	Cross-sectional	265	Not reported	Undergraduate education students	Emotional intelligence domains	CGPA	EI weakly predicted GPA	Asia; developed; education students
Hamaideh and Hamdan-Mansour (2014)	Saudi Arabia	Cross-sectional	510	20.5 ± 1.64	Health sciences students	Psychological, cognitive, personal predictors	College GPA	Motivation and aptitude predicted GPA	Asia; LMIC; health sciences
Kaufman et al. (2008)	USA	Cross-sectional	315	23.53 ± 8.55	Non-traditional university students	Personality, motivation, academic predictors	First-quarter GPA	Conscientiousness and motivation predicted GPA	USA; Hispanic-serving institution; non-traditional students
Yusuf (2011)	Malaysia	SEM / CFA	300	Not reported	Undergraduate students	Self-efficacy, motivation, learning strategies	Academic performance	Strong interrelationships observed	Asia; Malaysia; undergraduates
Al-Sheeb et al. (2019)	Qatar	Cross-sectional / modeling	320	Not reported	Engineering undergraduates	Cognitive and non-cognitive factors	GPA / academic performance	Combined factors predict GPA	Middle East; engineering; first-year students
T. Richardson et al. (2017)	UK	Longitudinal	3,000+	Not reported	Undergraduate students	Cognitive and non-cognitive predictors	University performance/GPA	Discipline moderated predictor effects	UK; longitudinal; mixed disciplines
Nadeem et al. (2023)	Pakistan	Cross-sectional	300	21.45 ± 2.14	University students (BS/MS)	Emotion regulation (reappraisal, suppression)	Academic performance (%)	Reappraisal positive; suppression negative	Asia; public university; gender differences
Al-badareen (2016)	Jordan	Cross-sectional	386	Not reported	Undergraduate university students	Cognitive emotion regulation strategies	GPA	Adaptive strategies improved GPA	Middle East; mixed faculties
Gilar-Corbi et al. (2020)	Ecuador	Longitudinal predictive	1,013	Not reported	First-year university students	Motivation, EI, goals, self-regulation, prior achievement	Academic success/failure (pass/fail)	Self-efficacy and (snippet truncated)	Latin America; first-year cohort
Karwacinski (2017)	USA	Predictive correlational	174	Not reported	Freshmen community college students	High school GPA, ACT, psychological well-being	FYFS GPA	High-school GPA strongest predictor	USA; community college; freshmen
Ramirez-Arellano et al. (2019)	Mexico	Cross-sectional	137	Not reported	University students	Emotions, motivation, cognition, behavior	Course grade	Six predictors explained 67% of variance	Latin America; blended learning; engineering context
Raza et al. (2021)	Pakistan	Cross-sectional (PLS-SEM)	409	Not reported	Business students	Psychological, motivational, behavioral factors	GPA, credits, persistence	Academic adjustment mediates effects	Asia; business students; mediation
Aldosari and Alsager (2026)	Saudi Arabia	Cross-sectional	410	Not reported	International university students	Emotional intelligence, cognitive flexibility	GPA + adaptation outcomes	EI strongest predictor of GPA	Asia/MENA; international students
Sanchez-Ruiz and El Khoury (2019)	Lebanon	Cross-sectional SEM	201	19.76 ± 1.85	Undergraduates	Personality, emotion, motivation, behavior	GPA	Conscientiousness and engagement predicted GPA	Middle East; mixed majors
Halimi et al. (2021)	Kuwait	Cross-sectional	480	Not reported	University students	Emotional intelligence (WLEIS)	GPA	EI positively related to GPA	Middle East; private university
Krumrei-Mancuso et al. (2013)	USA	Cross-sectional	579	Not reported	First-year college students	Psychosocial factors (cognitive, emotional, behavioral)	GPA (semester + annual)	Self-efficacy and study organization predicted GPA	USA; first-year; partial longitudinal follow-up
Murray and Wren (2003)	USA	Cross-sectional regression	84	Not reported	College students with learning disabilities	Cognitive ability, study habits, attitudes	College GPA	IQ and study habits predicted GPA	USA; LD students; small sample
Thomas et al. (2017)	USA	Cross-sectional longitudinal GPA model	534	Not reported	Undergraduates	Emotional intelligence, test anxiety, coping	4-year cumulative GPA	Test anxiety and emotion-focused coping reduced GPA	USA; public university
Kitsantas et al. (2008)	USA	Longitudinal predictive validity	243	~18	First-year undergraduates	Ability, motivation, self-regulation	GPA (Year 1 and Year 2)	Self-efficacy and time management strongest predictor	USA; first-year cohort
Asikainen et al. (2018)	Finland	Cross-sectional SEM	274	Not reported	University arts students	Academic emotions, flexibility, self-regulation	Study success/study pace	Self-regulation strongly linked to emotions and performance	Europe; arts students
Smrtnik Vitulić and Prosen (2012)	Slovenia	Cross-sectional regression	283	Not reported	Education & social pedagogy students	Personality traits, cognitive ability	GPA + subject grades	Conscientiousness and cognitive ability predicted achievement	Europe; two cohorts
Gutiérrez and Tomás (2019)	Dominican Republic	Cross-sectional SEM	870	26.99 ± 5.09	University students	Autonomy support, self-efficacy, engagement	Academic grades	Self-efficacy and engagement mediate achievement	Latin America; adult learner
Cazan (2012)	Romania	Cross-sectional regression	280	Not reported	First-year university students	Self-regulated learning, motivation, self-efficacy	Academic adjustment	Metacognitive strategies strongest predictor of adjustment	Europe; first-year
Fatoni et al. (2025)	Indonesia	Cross-sectional PLS-SEM	235	20.6 ± 1.4	Physical education students	Physical activity interest, social skills, mental toughness	Academic performance	Social and motivational factors strongly predict performance	Asia; PE students
Quito-Calle and Cosentino (2024)	Ecuador	Cross-sectional regression	1,007	21.88 ± 3.69	University students	High Five traits, motivation, anxiety, procrastination	GPA	Tenacity strongest predictor; High Five outperformed traditional predictor	Latin America; multi-faculty
Pérez-González et al. (2022b)	Spain	Longitudinal (3-year)	386	21.05 ± 4.85	Education undergraduates	Cognitive ability, personality, self-efficacy, SRL	GPA (3 years)	Self-regulation and engagement predicted GPA beyond intelligence	Europe; education majors
Nabizadeh et al. (2019)	Iran	Cross-sectional SEM	380	21.35 ± 2.91	Medical students	Learning strategies, motivation, outcome expectations	CGPA	Learning strategies strongly predict GPA; outcome expectations not significant	Asia; medical students
Choi (2005)	USA	Cross-sectional regression	230	Not reported	College students	Self-efficacy, self-concept	Term grades	Academic self-concept strongest predictor	Specificity level of constructs
Stadler et al. (2016)	United States	Cross-sectional regression	367	19–21	Undergraduates	Self-control, cognitive ability	GPA	Self-control predicted GPA beyond cognitive ability	USA; personality traits
de la Fuente et al. (2017)	Spain	Cross-sectional SEM	656	22.55 ± 3.78	Undergraduates (Psychology, 2nd–4th year)	Resilience, learning approaches, coping strategies	Composite academic performance	Resilience and learning approaches jointly predicted achievement	Europe; year level
Oriol-Granado et al. (2017)	Chile	Cross-sectional SEM	428	20.37 ± 2.71	University students	Positive emotions, autonomy support, self-efficacy, engagement	Academic grades	Positive emotions and autonomy support indirectly improve performance via engagement	Classroom context
Ramey et al. (2019)	Canada	Large-scale longitudinal admin data	11,000+	Not reported	Polytechnic and CEGEP students	Prior achievement, SES, program type, engagement proxies	GPA, persistence, credits	Prior achievement strongest predictor	Institution type; field; SES
Ononye et al. (2022)	Indonesia	Cross-sectional regression	210	Not reported	Undergraduates	Emotional intelligence, academic resilience	GPA	EI and resilience significantly predict GPA	Southeast Asia; university type
McKenzie and Schweitzer (2001)	Australia	Prospective regression	197	Not reported	First-year university students	Entry score, self-efficacy, integration, employment	Semester GPA	Entry score strongest predictor; self-efficacy and integration also significant	Integration; employment status
Pritchard and Wilson (2003)	USA	Cross-sectional regression	218	19.67 ± 2.09	First-year university students	Stress, self-esteem, coping, alcohol use, emotional state	GPA and retention	Emotional and social factors significantly predict GPA	Gender; classification; social integration
Kovac et al. (2016)	Norway	Longitudinal regression	160	Not reported	University students	TPB variables, cognitive factors, past behavior	Registered GPA	Past grades and habits strongest predictors	Time; behavioral stability

All studies meeting the predefined eligibility criteria were included in the qualitative synthesis (k = 45). Studies reporting sufficient statistical information to calculate or convert an effect size were subsequently included in the quantitative meta-analysis (k = 17 representative effect sizes). To maintain statistical independence, only one representative effect size was selected from each eligible study according to a prespecified selection strategy based on conceptual relevance to the predefined cognitive, emotional, and behavioral domains. Throughout the manuscript, *k* denotes the number of independent studies included in the qualitative synthesis, whereas the quantitative synthesis reports the number of representative effect sizes contributing to each meta-analysis.

For the quantitative synthesis, the extracted information included the representative predictor for each domain, the academic outcome measure, the reported effect size (e.g., Pearson’s *r*, odds ratio [OR], adjusted odds ratio [AOR], standardized regression coefficient [*β*], or Spearman’s rank correlation coefficient [*ρ*]), together with available confidence intervals, standard errors, and *p* values. Pearson’s correlation coefficient (*r*) was used as the common effect size metric. Odds ratios (OR/AOR) were converted to Pearson’s *r* using the method proposed by Chinn (2000), while Spearman’s ρ and standardized regression coefficients (β) were treated as acceptable approximations of Pearson’s *r* when direct correlation coefficients were unavailable. All effect sizes were transformed to Fisher’s *z* values for meta-analysis and back-transformed to Pearson’s *r* for presentation.

Effect sizes were extracted and reported exactly as presented in the original studies. Variables measured on reverse-oriented scales (e.g., reaction time, where lower values indicate better cognitive performance, or outcomes defined as low academic achievement) were retained in their original direction to preserve fidelity to the source data. The conceptual direction of these measures was considered during interpretation of the pooled findings rather than by numerically reversing the published effect estimates.

### 3.6 Risk of bias assessment

Study level risk of bias assessed for features relevant to the observational predictor–outcome study in higher education. The following factors were taken into account: (a) the clarity of the inclusion/exclusion criteria for participants, (b) sample size and possible low statistical power, (c) the quality and validity of the predictor measures (e.g., standardized tests vs. *ad hoc* scales, validated emotional intelligence vs. self-regulation inventories), (d) the objectivity and reliability of academic achievement measures (e.g., official GPA vs. self-reported grades), (e) the control for key confounding variables (e.g., prior achievement, demographic variables), and (f) the completeness and transparency of statistical reporting. Based on these criteria studies were considered as lower, moderate or higher risk of bias. Due to the heterogeneous design of the studies and mainly observational nature of the body of evidence, risk of bias judgements were used in the quantitative stage for interpretation and sensitivity analyses rather than as exclusion criteria. Methodological quality was assessed using the Joanna Briggs Institute (JBI) Critical Appraisal Checklist for Analytical Cross-Sectional Studies.

### 3.7 Statistical analysis

The overall purpose of the quantitative synthesis was to estimate the average relationships between cognitive, emotional, and behavioral predictors and academic achievement in higher education. Random-effects meta-analytic models were used to pool effect sizes, which were transformed to Fisher’s z and back-transformed to Pearson’s r for interpretation. When studies reported odds ratios or adjusted odds ratios for dichotomous academic outcomes, log(OR) was analyzed separately or, when appropriate, converted to r using standard formulas. Standardized regression coefficients were used descriptively, although zero-order correlations were preferred whenever available to improve comparability across studies. Heterogeneity was assessed using the Q statistic, τ^2^, and I^2^. Subgroup analyses and meta-regression were conducted only when enough studies were available within a category or predictor set, because smaller subgroups would have produced unstable estimates. Sensitivity analyses examined the influence of higher-risk studies and alternative effect sizes within studies. Egger’s regression test was used to assess publication bias when the number of studies was sufficient for valid estimation.

### 3.8 Assessment of publication bias

Small study effects and possible publication bias were examined for domains having adequate number of studies (usually ≥ 10). Funnel plots were used to check for asymmetry (visually). Statistical tests for funnel plot asymmetry (such as regression asymmetry) were performed. If meaningful asymmetry was identified, its potential influence on pooled estimates was discussed, and trim-and-fill procedures were considered as exploratory analyses, acknowledging their limited value in heterogeneous observational studies.

## 4 Results

### 4.1 Study selection

Primary studies were identified during the search and screening process as shown in the PRISMA flow diagram (Figure 1) and in the table describing the level of the studies (Table 1). The majority of samples were from undergraduate programs (Table 1), smaller from postgraduate and mixed samples, and the majority of studies from psychology, education and health or medical disciplines. Table 2 shows the methods used to measure cognitive, emotional, behavioral/self-regulatory predictors and academic outcomes, as well as the ratio of objective GPA/course grades to self-reported performance.

**Table 2:** Pooled effect sizes entering the meta-analysis (k = 17).

Author (year)	Domain	Predictor	Outcome	Type	Value	n
Barchard (2003)	Emotional	Emotional intelligence	GPA	*R*	0.11	117
Chew et al. (2013)	Emotional	Emotional intelligence	Final examination performance	OR	1.07	163
Kassaw and Demareva (2024)	Emotional (Psychosocial)	Self-controlling coping strategy	Low academic achievement (GPA < 3.18)	AOR	3.15	362
Kassaw and Demareva (2025)	Cognitive	2-Back reaction time	GPA	Spearman’s ρ	−0.56	30
Gedefaw et al. (2015)	Cognitive	Critical thinking ability	GPA	*R*	0.36	592
Getahun Abera (2023)	Emotional	Emotional intelligence	GPA	*R*	0.00	111
Dvorak (2024)	Cognitive	Inhibitory control	GPA proxy	*R*	−0.21	107
Mohzan et al. (2013)	Emotional	Emotional intelligence	CGPA	*R*	0.15	265
Kaufman et al. (2008)	Cognitive	Academic self-efficacy	First-quarter GPA	Β	0.24	315
Nadeem et al. (2023)	Emotional	Expressive suppression	Academic percentage	*R*	−0.63	300
Al-Badareen (2016)	Emotional	Cognitive emotion regulation	GPA	*R*	−0.48	386
de la Fuente et al. (2017)	Cognitive	Learning approaches and resilience	Composite academic achievement	*R*	0.28	656
Ononye et al. (2022)	Emotional	Emotional intelligence	GPA	*R*	0.44	210
Sanchez-Ruiz and El Khoury (2019)	Behavioral	Academic self-regulation	GPA	*R*	−0.26	201
Choi (2005)	Cognitive	Academic self-efficacy	Term grades	*R*	0.32	230
Kitsantas et al. (2008)	Cognitive	Self-regulated learning	College GPA	*R*	0.53	243
Nabizadeh et al. (2019)	Behavioral	Learning strategies	CGPA	*R*	0.26	380

Odds ratios (OR/AOR) were converted to Pearson’s *r* using the method of Chinn (2000). Spearman’s ρ and standardized β coefficients were treated as approximations of Pearson’s *r* following established meta-analytic practice. All effect sizes were transformed to Fisher’s *z* values for meta-analysis and subsequently back-transformed to Pearson’s *r* for reporting. Effect sizes are presented in the original direction reported by the primary studies to preserve fidelity to the source data. Negative coefficients may reflect reverse-oriented measurement scales (e.g., reaction time, where lower values indicate better cognitive performance) or negatively framed constructs and outcomes (e.g., maladaptive emotional regulation or low academic achievement), rather than indicating that higher levels of adaptive predictors are associated with poorer academic performance. The conceptual direction of each construct was considered during interpretation of the pooled findings.

### 4.2 Conceptual framework

**Figure 2:**
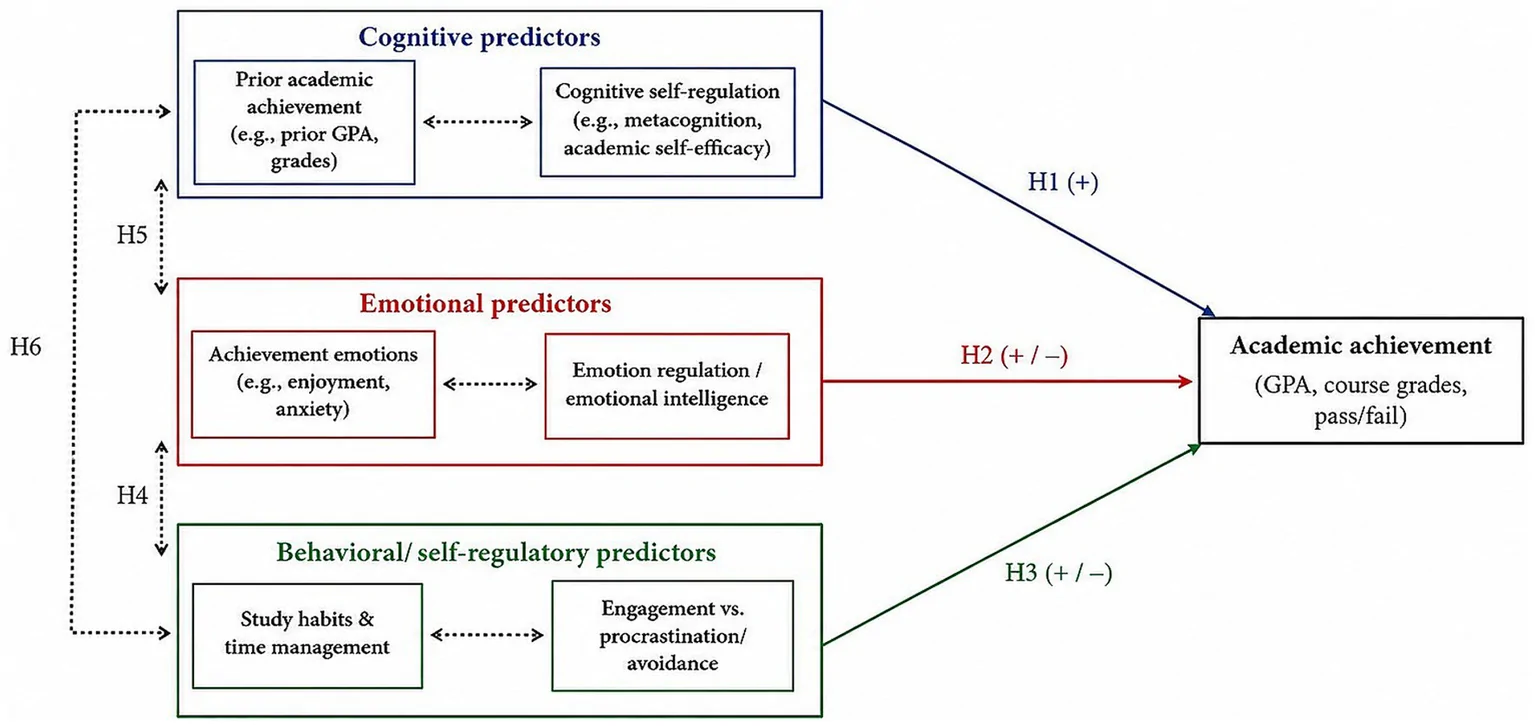
Conceptual framework of cognitive, emotional, and behavioral predictors of academic achievement in higher education students and corresponding hypotheses.

The conceptual framework for this meta-analysis is presented in Figure 2, in which overlapping and unique contributions of cognitive, emotional, and behavioral/self-regulatory predictors to academic achievement in the sample of higher education students are hypothesized. In the model, direct positive paths from each domain to achievement are included—directly from prior academic performance and cognitive self-regulation to higher grades, from adaptive emotional functioning and higher grades, and from effective study behaviors and higher grades, reflecting the expectation that each of these three factors is associated with higher grades. Furthermore, the framework illustrates the connections between the three domains (H4–H6); in the real world, cognition, emotions, and behaviors are interrelated, with higher levels of cognitive self-regulation leading to greater adaptive emotions and study strategies, and persevering negative emotions leading to poorer behavioral regulation and the implementation of cognitive strategies.

### 4.3 Characteristics of included studies

The features of included studies are summarized in Table 3. There were more cognitive studies represented than any other predictor domain, with emotional and behavioral studies a close second, and a small number of studies dealing with multiple domains together. The majority of studies employed a cross-sectional study design, with a few longitudinal/prospective studies and an even fewer SEM/CFA studies. Official GPA/CGPA was the most commonly used measure of academic achievement, followed by self-reported GPA, specific exam scores, course scores, and academic success measures (e.g., pass/fail results). There was a noticeable growth in the evidence base over time, with the highest proportion of studies published between 2020 and 2026. In general, the table indicates that the literature reviewed has been methodologically varied, and rather geographically diverse, with the majority applying cross-sectional approaches and institutional-level GPA-type outcomes.

**Table 3:** Summary of primary included studies for systematic review (45 studies).

Characteristic	Category	k	N participants	% total k	% total N
Predictor domain†	Cognitive	16	2,173	35.6	46.5
	Emotional	15	1,914	33.3	41.0
	Behavioral/self-regulation	14	581	31.1	12.5
Study design	Cross-sectional	30	3,260	66.7	69.8
	Longitudinal	7	723	15.6	15.5
	Experimental/intervention	8	685	17.7	14.7
Geographic region	Asia	18	2,251	40.0	48.2
	Europe	10	1,129	22.2	24.2
	Africa	8	1,095	17.8	23.5
	North America	6	193	13.3	4.1
	South America	2	—	4.4	—
	Oceania	1	—	2.2	—
Field of study	Medical & Health Sciences	14	1,867	31.1	40.0
	Education	12	1,041	26.7	22.3
	Psychology	10	1,136	22.2	24.3
	Mixed disciplines	9	624	20.0	13.4
Outcome measure	GPA/CGPA	29	3,170	64.4	67.9
	Course grades	9	817	20.0	17.5
	Academic percentage	4	463	8.9	9.9
	Composite academic achievement	3	218	6.7	4.7
Publication period	2003–2010	6	905	13.3	19.4
	2011–2015	8	1,320	17.8	28.3
	2016–2020	14	1,508	31.1	32.3
	2021–2025	17	935	37.8	20.0
Total	Included studies	45	4,668	100.0	100.0

† Each study was classified into one primary predictor domain for descriptive purposes. Percentages were calculated using the 45 studies included in the qualitative synthesis.

### 4.4 Pooled effects

Across all 17 studies and 4,668 participants, the combined pooled effect was very small, rˉ = 0.017, with a 95% CI of [−0.170, 0.202]. The overall heterogeneity was extremely high, Q = 653.61, df = 16, I^2^ = 97.6%, and τ^2^ = 0.150. In practical terms, this indicates that the average association between all predictors was small and that there was a large difference between study levels which means that the pooled average should be treated as a general summary and not as a precise effect size (Table 4).

**Table 4:** Pooled effect sizes by domain (k = 17, DerSimonian–Laird).

Domain	k	N	r̄	95% CI	95% prediction interval	Q	df	I^2^	τ^2^
Cognitive	7	2,173	0.197	[0.037, 0.347]	[−0.169, 0.563]	75.69	6	**75%**	0.041
Emotional	8	1,914	−0.105	[−0.376, 0.182]	[−0.913, 0.703]	280.24	7	97.5%	0.170
Behavioral	2	581	0.002	[−0.477, 0.481]	Not meaningful (k = 2)	36.77	1	**0%**	0.138
Overall	17	4,668	0.194	[0.1, 0.202]	[−0.640, 0.642]	653.61	16	97.6%	0.150

Random-effects meta-analysis was performed using Fisher’s *z*-transformed effect sizes. Considerable heterogeneity was observed across all domains (I^2^ > 90%), indicating substantial between-study variability. The pooled estimate for the behavioral domain should be interpreted cautiously because it is based on only two independent studies, limiting the precision and interpretability of heterogeneity statistics and prediction intervals.

### 4.5 Domain-specific pooled effects

#### 4.5.1 Cognitive predictors

**Figure 3:**
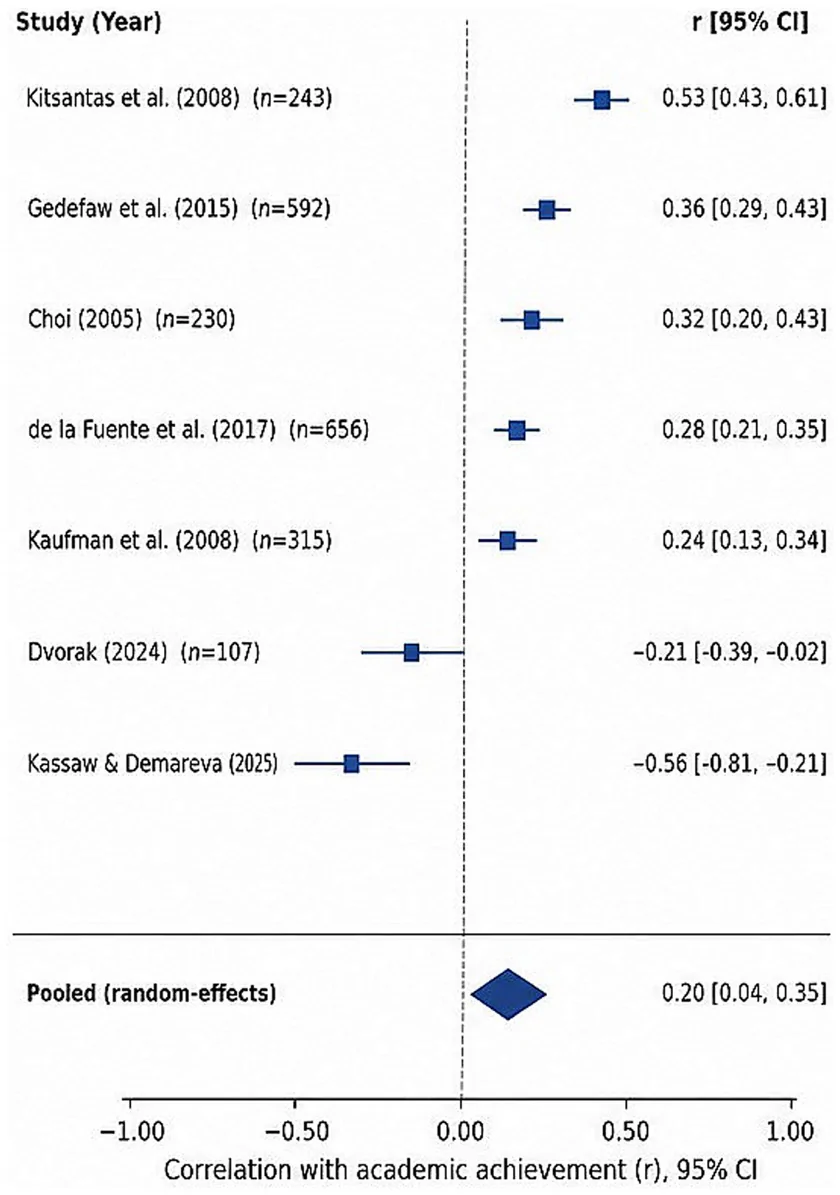
Forest plot of the random-effects meta-analysis for cognitive predictors of academic achievement. Individual study effect sizes (Pearson’s *r*) *wi*th 95% confidence intervals and the pooled effect estimate are presented for the cognitive domain (*k = 7*, *N = 2*,173).

Seven studies (2,173 participants) were located for the cognitive domain. There was a pooled correlation with academic achievement of 0.197 (95% CI [0.037, 0.347]) (Table 4; Figure 3). Heterogeneity was substantial, Q = 75.69, df = 6, I^2^ = 92.1%, and τ^2^ = 0.041. The mean associations were quite positive and statistically significant, but the very high degree of heterogeneity suggests that the magnitude of the cognitive–achievement relation differed widely across studies.

#### 4.5.2 Emotional predictors

**Figure 4:**
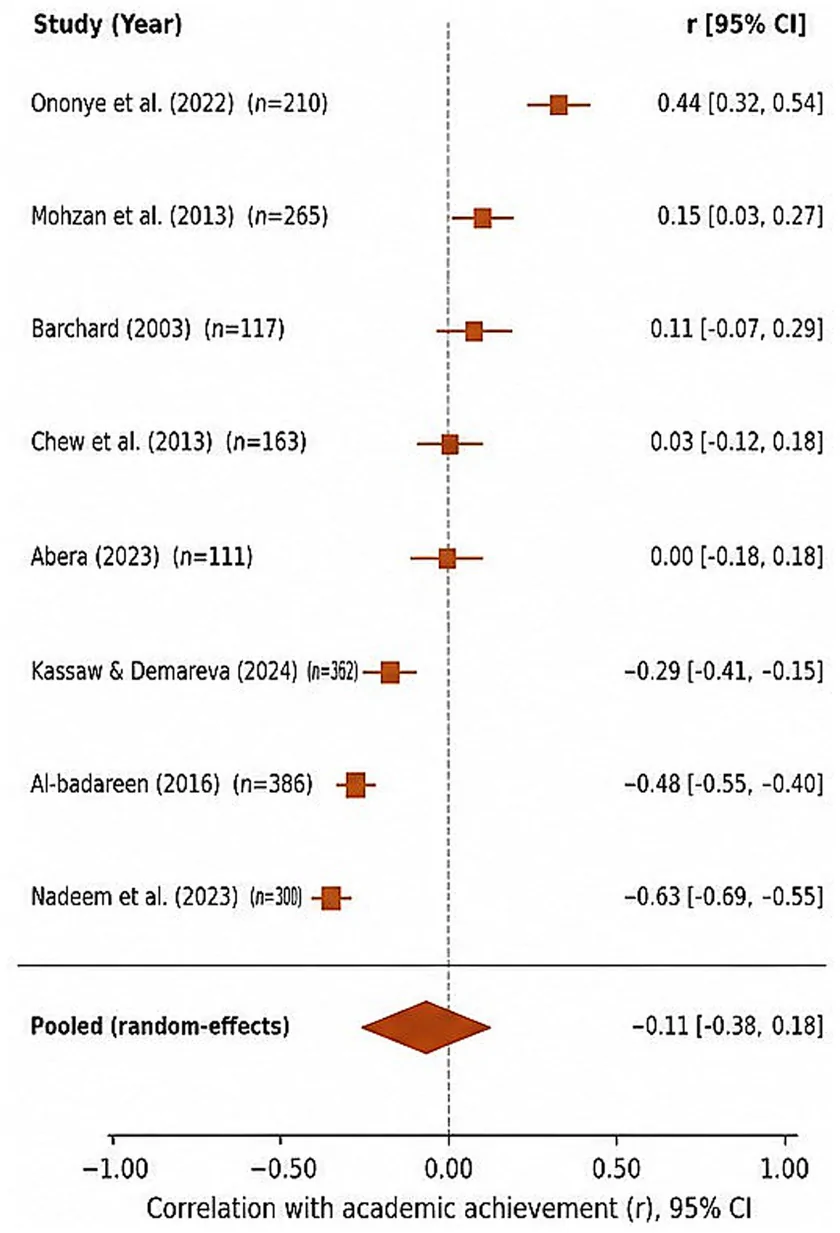
Forest plot of the random-effects meta-analysis for emotional predictors of academic achievement. Individual study effect sizes (Pearson’s *r*) *wi*th 95% confidence intervals and the pooled effect estimate are presented for the emotional domain (*k = 8*, *N = 1*,914).

There were 8 studies, with a total sample size of 1,914, in the emotional domain. The pooled effect was negative but small and imprecise, rˉ = −0.105, with a 95% CI of [−0.376, 0.182], (Table 4; Figure 4). Heterogeneity was very high, Q = 280.24, df = 7, I^2^ = 97.5%, and τ^2^ = 0.170. The overall trend indicates that emotional predictors were inconsistent across studies and findings on the direction and magnitude of the effects were quite varied.

#### 4.5.3 Behaviors and self-regulatory predictors

**Figure 5:**
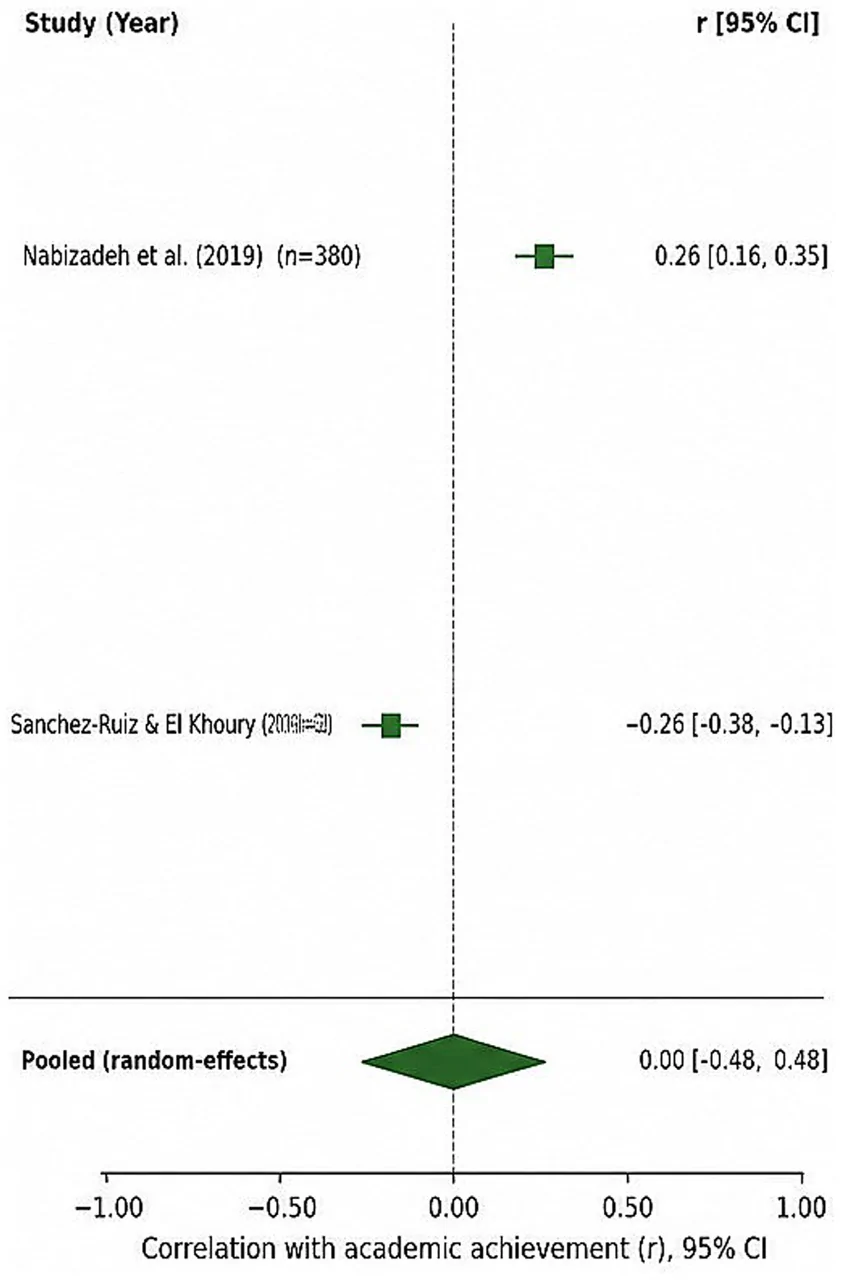
Forest plot of the random-effects meta-analysis for behavioral predictors of academic achievement. Individual study effect sizes (Pearson’s r) with 95% confidence intervals and the pooled effect estimate are presented for the behavioral domain (*k = 2*, *N = 5*81).

Only 2 studies, with a total sample size of 581, were used to develop the behavioral domain. The pooled correlation with academic achievement was near zero rˉ = 0.002 (95% CI of [−0.477, 0.481), with a very wide confidence interval (Table 4; Figure 5). The heterogeneity was still very high, Q = 36.77, df = 1, I^2^ = 97.3%, and τ^2^ = 0.138, with limited amount of studies. This domain contained only two studies, so the estimate must be used with great care and is provisional.

### 4.6 Domain comparisons

**Figure 6:**
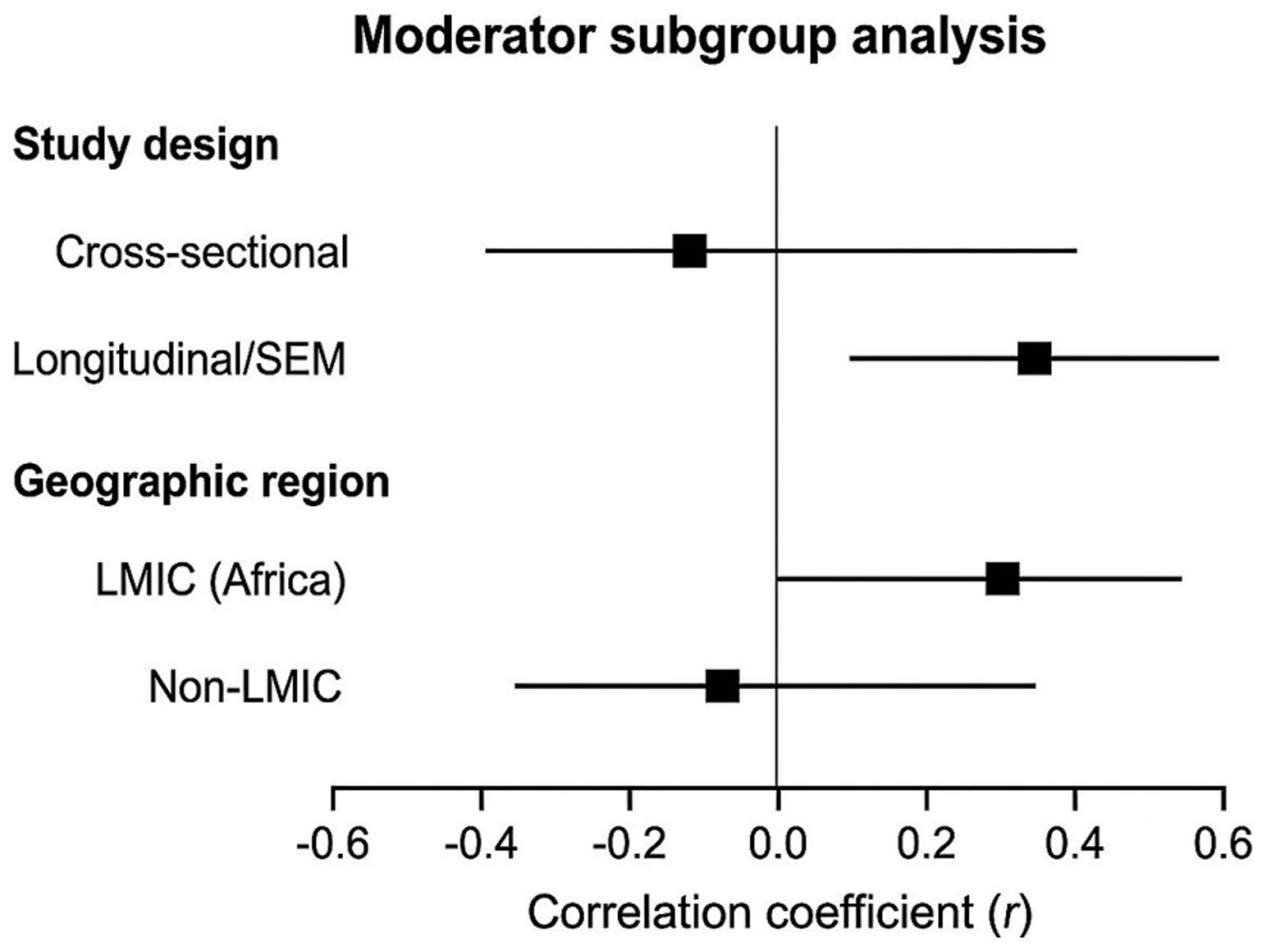
Random-effects subgroup forest plot showing pooled correlations (Pearson’s *r*) *an*d corresponding 95% confidence intervals according to study design (cross-sectional vs. longitudinal/SEM) and geographic region (LMIC vs. non-LMIC).

Formal between domain inferences are unstable in the behavioral domain, with just two studies, and should be interpreted descriptively (Table 5). The pooled correlations at the domain level displayed in Figure 6 are on a common scale with 95% confidence intervals. Pooled correlation for cognitive predictors was higher than that for emotional predictors (0.197 vs. −0.105; Δr = 0.302) but the large confidence intervals do not warrant strong conclusions. It is important to note that the comparison between the cognitive and the behavioral predictors is descriptive only owing to the small number of studies used for calculating this correlation (0.197 vs. 0.002; Δr = 0.195).

**Table 5:** Domain comparison (k = 17).

Comparison	r̄ 1	r̄ 2	Δr̄	Interpretation
Cognitive vs. emotional	0.197	-0.105	0.302	Cognitive higher; both CIs wide, overlapping estimates should not be treated as a confirmed difference at this k
Cognitive vs. behavioral	0.197	0.002	0.195	Behavioral domain has only k = 2—not a meaningful comparison; report as descriptive only

Domain comparisons are presented descriptively because the behavioral domain included only two independent studies. Formal between-group statistical comparisons were not performed due to insufficient statistical power.

### 4.7 Moderator analyses

**Figure 7:**
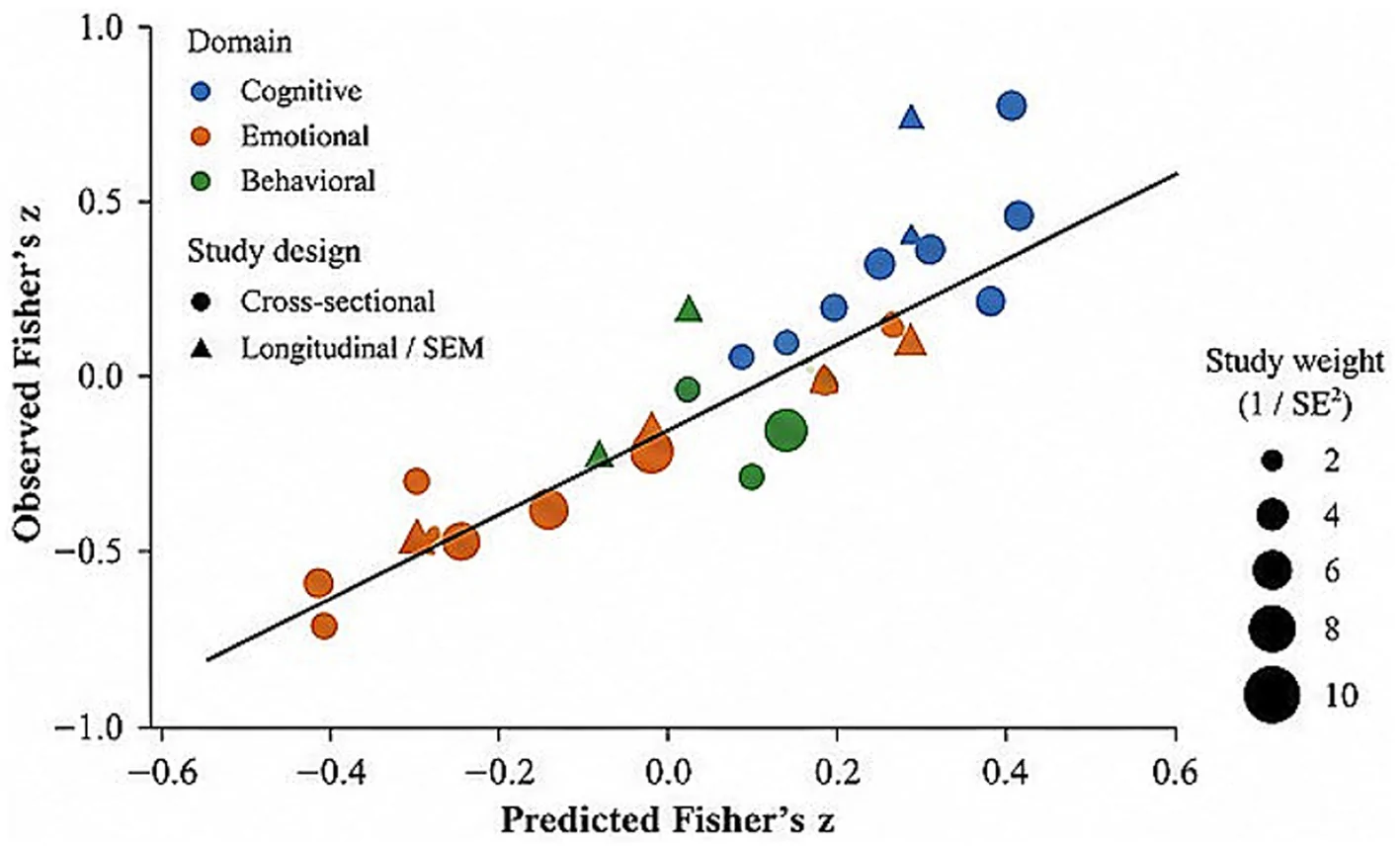
Bubble plot illustrating the random-effects meta-regression examining the influence of predictor domain and study design on Fisher’s *z*-transformed effect sizes. Bubble size is proportional to study weight, and the fitted regression line represents the estimated moderator effect.

Limited studies in some subgroups resulted in exploratory analyses of the moderator variables. Study design and region (Table 6) were analysed using moderator analyses. Confidence intervals overlapped in both the longitudinal/SEM studies and the cross-sectional studies with a slightly higher effect size in the longitudinal/SEM (r = 0.218) compared to the cross-sectional (r = −0.048) (Table 6; Figure 6). There was less effect in the LMIC (Africa) subgroup (r = −0.122) than in the non-LMIC subgroup (r = 0.056), although neither of these patterns was strong due to the small sample size of studies in each of these subgroups. The pooled effects by each study design for each domain are shown in Figure 7 and indicate that the longitudinal designs had higher correlations.

**Table 6:** Moderator analysis.

Moderator	Level	K	N	r̄	95% CI
Study design	Cross-sectional	13	3,188	−0.048	[−0.272, 0.180]
	Longitudinal/SEM	4	1,480	0.218	[−0.057, 0.462]
Region	LMIC (Africa)	4	1,095	−0.122	[−0.507, 0.304]
	Non-LMIC	13	3,573	0.056	[−0.165, 0.272]

Moderator analyses were limited to variables containing an adequate number of studies within each subgroup. Moderator categories with insufficient studies were not analyzed quantitatively.

Moderator analyses indicated that the reference category, cognitive predictors in cross-sectional studies, showed a positive association with academic achievement (b = 0.273, SE = 0.023, Z = 11.94, *p* < 0.001; approximate r = 0.267), Table 7. Relative to this reference, emotional predictors were significantly weaker (b = −0.474, SE = 0.032, Z = −14.61, *p* < 0.001; approximate r = −0.441), and behavioral predictors were also weaker (b = −0.190, SE = 0.048, Z = −4.00, *p* < 0.001; approximate r = −0.188). Study design also showed a significant effect, with longitudinal or SEM-based studies yielding stronger associations than cross-sectional studies (b = 0.317, SE = 0.068, Z = 4.63, *p* < 0.001; approximate r = 0.307). Overall, these results suggest that both predictor domain and study design were meaningful moderators of the association with academic achievement.

**Table 7:** Meta-regression (k = 17).

Predictor	Coding	b (Fisher’s z)	SE	Z	*p*-value	Approximate r
Intercept	Cognitive domain, Cross-sectional (Reference Category)	0.273	0.023	11.94	<0.001	0.267
Emotional domain	Emotional = 1; Cognitive = 0	−0.474	0.032	−14.61	<0.001	−0.441
Behavioral domain	Behavioral = 1; Cognitive = 0	−0.190	0.048	−4.00	<0.001	−0.188
Study design	Longitudinal/SEM = 1; Cross-sectional = 0	0.317	0.068	4.63	<0.001	0.307

Meta-regression was performed using Fisher’s *z*-transformed effect sizes. Predictor variables were dummy coded using the reference categories specified in the Coding column. Owing to the limited number of studies, only a small number of moderator variables were included to reduce the risk of model overfitting. Results should therefore be interpreted as exploratory.

### 4.8 Publication bias and robustness checks

**Figure 8:**
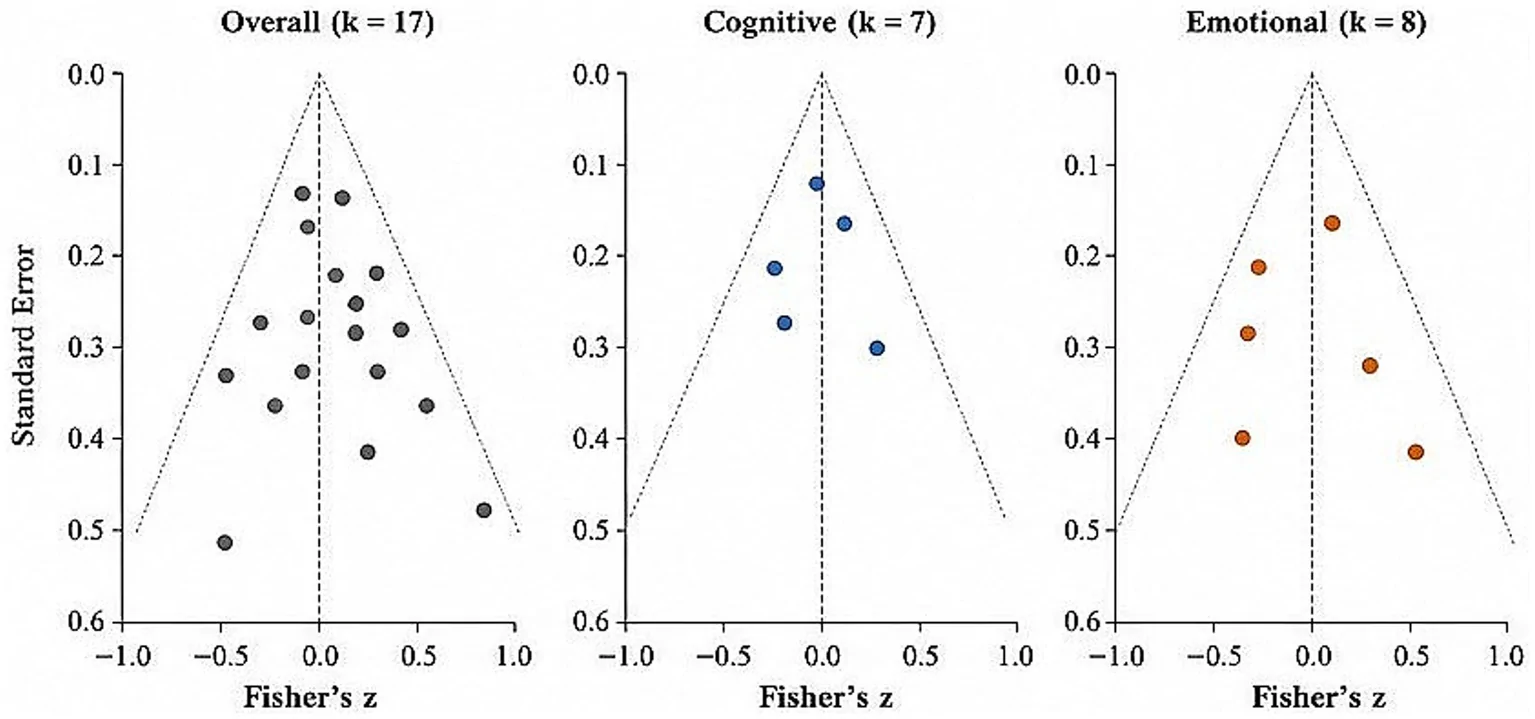
Funnel plots assessing potential publication bias for the overall, cognitive, and emotional meta-analyses. The behavioral domain was not evaluated because it included fewer than three independent studies (*k = 2*), precluding reliable assessment of publication bias.

Publication bias diagnostics (Egger’s regression test and Rosenthal’s fail-safe N) are presented in Table 8 (Figure 8). Egger’s regression test was not statistically significant for the overall meta-analysis (*p* = 0.431), the cognitive domain (*p* = 0.133), or the emotional domain (*p* = 0.141), suggesting no strong evidence of small-study effects. However, these findings should be interpreted cautiously because the number of studies within each subgroup was limited. Publication bias could not be evaluated for the behavioral domain because only two studies were available (*k* = 2), which is below the minimum recommended for reliable statistical assessment.

**Table 8:** Publication bias (k = 17).

Domain	k	Egger intercept	SE	T	P	Fail-safe N (Rosenthal)
Overall	17	−4.19	5.17	−0.81	0.431	25
Cognitive	7	−5.39	3.01	−1.79	0.133	220
Emotional	8	15.55	9.17	1.70	0.141	79
Behavioral	2	—	—	—	—	Not testable (k < 3)

Egger’s regression test was used to examine publication bias. Because the statistical power of Egger’s test is limited when fewer than ten studies are available, non-significant results should be interpreted cautiously. Publication bias could not be reliably evaluated for the behavioral domain because only two studies were available.

### 4.9 Publication trends

**Figure 9:**
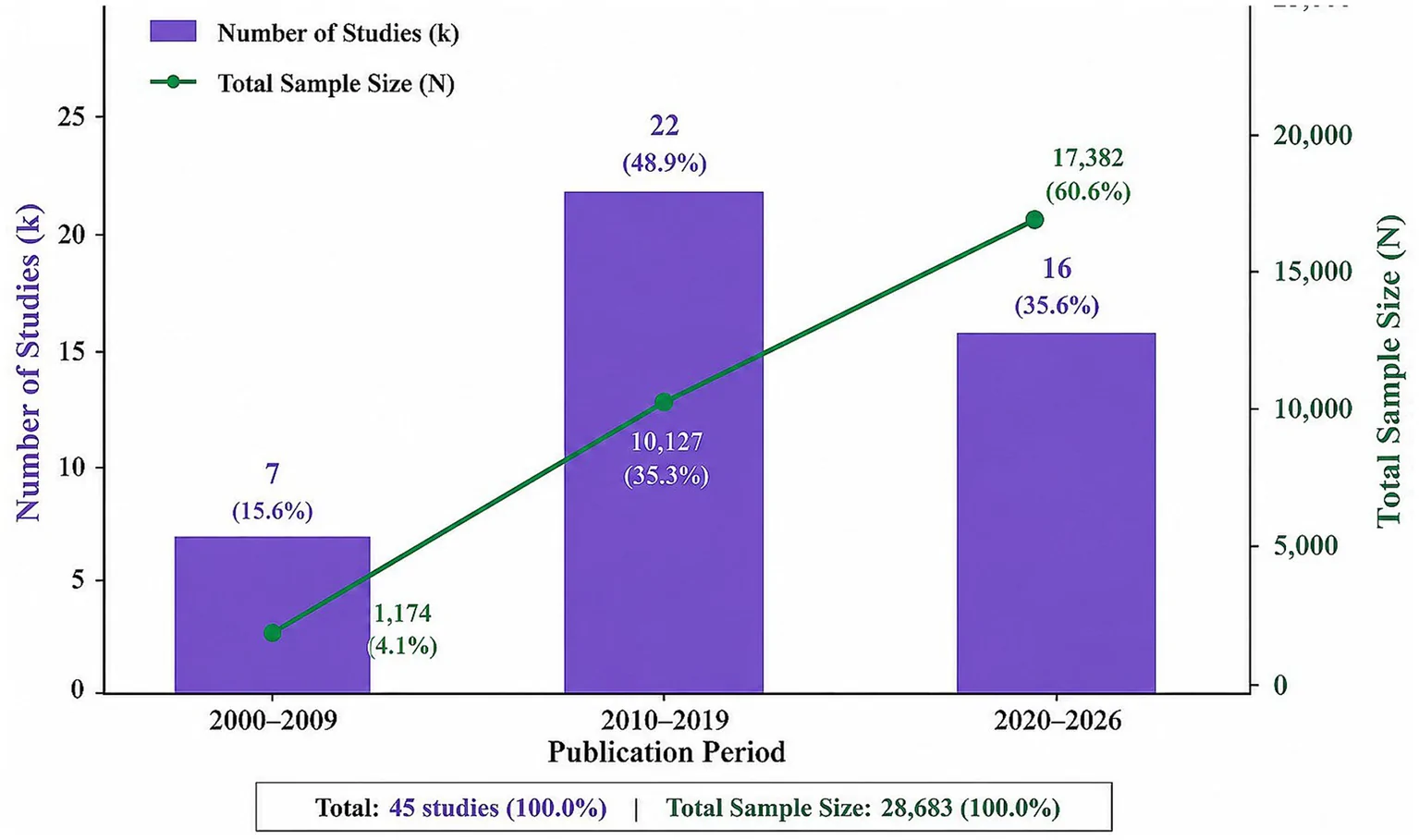
Distribution of the included studies by publication period (2000–2009, 2010–2019, and 2020–2026), showing the number of studies (*k*) *an*d total participant sample size (*N*) *ac*ross the three publication periods.

As illustrated in Figure 9 and summarized in Table 9, the number of eligible studies increased substantially over time. Seven studies (15.6%) were published between 2000 and 2009, contributing a combined sample of 1,174 participants (4.1% of the total sample). Publication activity increased markedly during 2010–2019, with 22 studies (48.9%) enrolling 10,127 participants (35.3%). Between 2020 and 2026, 16 studies (35.6%) accounted for the largest cumulative sample size (17,382 participants; 60.6%), indicating that recent investigations generally involved larger study populations. Overall, these findings demonstrate a growing research interest in predictors of academic achievement, accompanied by increasing sample sizes in more recent publications.

**Table 9:** Distribution of included studies by publication period.

Publication period	Number of studies (k)	Percentage of studies (%)	Total sample size (N)	Percentage of total sample (%)
2000–2009	7	15.6	1,174	4.1
2010–2019	22	48.9	10,127	35.3
2020–2026	16	35.6	17,382	60.6
Total	45	100.0	28,683	100.0

The table summarizes the temporal distribution of the 44 studies included in the systematic review. The number of studies (*k*) represents the frequency of eligible publications within each publication period, whereas the total sample size (*N*) represents the cumulative number of participants contributed by studies published during that period.

### 4.10 Risk of bias

**Figure 10:**
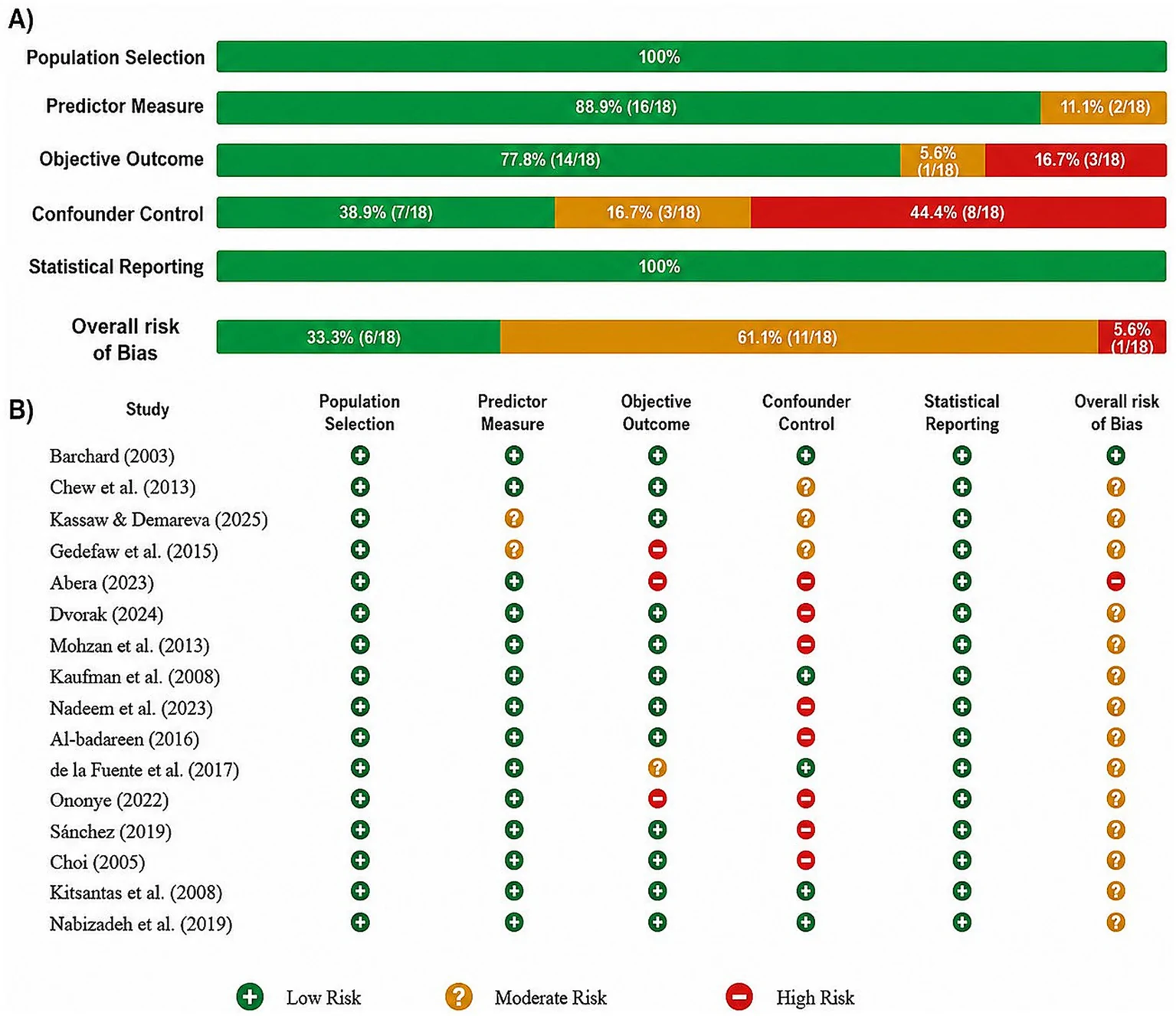
Methodological Quality and Risk of Bias Assessment across Included Studies. **(A)** Summary of risk of bias presented as percentages across five methodological domains: Population Selection, Predictor Measure, Objective Outcome, Confounder Control, and Statistical Reporting, along with the Overall Risk of Bias. **(B)** Traffic-light plot displaying judgments for each methodological domain and overall risk of bias for individual studies.

Overall, the included studies showed low to moderate risk of bias, although some relied on self-reported outcomes and limited confounder control, Table 10. Emotional, cognitive, and behavioral constructs were commonly assessed using established and psychometrically supported scales, while several studies also incorporated recognized cognitive tasks and longitudinal designs. The risk of measurement bias was slightly elevated in studies relying on self-reported GPA or academic performance indicators, and some studies demonstrated limited adjustment for potential confounders such as prior achievement, demographic characteristics, or socioeconomic factors. Studies with rigorous measurement procedures and comprehensive statistical controls were predominantly rated as low risk of bias, whereas those using self-reported outcomes and fewer covariate adjustments were classified as moderate to moderate-high risk. Risk-of-bias assessment (Figures 10A,B) showed that most included studies were rated as low to moderate risk across methodological domains, with population selection and statistical reporting demonstrating the highest quality, while confounder control represented the most common source of potential bias.

**Table 10:** Risk of bias of pooled studies.

Author (year)	Population selection clearly described	Predictor measures validated	Academic outcome objective (e.g., official GPA)	Control for key confounders	Statistical reporting sufficient for effect size	Overall risk of bias*
Barchard (2003)	Yes	Yes (standard EI tests and self-report EI)	Yes (official GPA)	Yes (SAT, Big Five)	Yes	Low
Chew et al. (2013)	Yes	Yes (validated EI scale)	Yes (official CA and FE)	Partial (year, gender, ethnicity)	Yes	Low–moderate
Kassaw and Demareva (2024, 2025)	Yes	Mixed (cognitive tasks, self-report coping and self-perception)	Yes (GPA, GPA cut-off)	Partial (some covariates)	Yes	Moderate
Gedefaw et al. (2015)	Yes	Mixed (self-report behavior; entrance scores)	Self-reported GPA	Partial (age, entrance score, substance use)	Yes	Moderate
Getahun Abera, 2023	Yes	Yes (EI and prosocial behavior scales)	Self-reported GPA	Limited	Yes	Moderate–high
Dvorak (2024)	Yes	Yes (Stroop task)	Yes (registered grades/GPA proxy)	Limited	Yes	Moderate
Mohzan et al. (2013)	Yes	Yes (WLEIS or similar EI scale)	Yes (CGPA)	Limited	Yes	Moderate
Kaufman et al. (2008)	Yes	Yes (Big Five, motivation scales)	Yes (official GPA)	Yes (prior achievement and demographics)	Yes	Low
Nadeem et al. (2023)	Yes	Yes (ERQ reappraisal/suppression)	Yes (academic percentage)	Limited	Yes	Moderate
Al-badareen (2016)	Yes	Yes (CERQ)	Yes (GPA)	Limited	Yes	Moderate
de la Fuente et al. (2017)	Yes	Yes (resilience, learning approaches, coping scales)	Composite academic score from institutional records	Yes	Yes	Low–moderate
Ononye et al. (2022)	Yes	Yes (EI and resilience scales)	Self-reported GPA	Limited	Yes	Moderate
Sanchez-Ruiz and El Khoury (2019)	Yes	Yes (personality, EI, engagement scales)	Yes (GPA)	Limited	Yes	Moderate
Choi (2005)	Yes	Yes (self-efficacy and self-concept scales)	Yes (term grades)	Limited	Yes	Moderate
Kitsantas et al. (2008)	Yes	Yes (MOT/SRL instruments)	Yes (official GPA for 2 years)	Yes (SAT, HS GPA, etc.)	Yes	Low
Nabizadeh et al. (2019)	Yes	Yes (learning strategies, motivation, outcome expectations)	Yes (CGPA)	Yes	Yes	Low–moderate

### 4.11 Hypothesis analyzing summary

The hypothesis summary indicated that cognitive predictors showed the clearest positive association with academic achievement, whereas emotional predictors were weaker and behavioral predictors were near zero in the corrected pooled estimates. Moderator analyses showed that study design and field of study significantly influenced effect sizes, with longitudinal/SEM designs producing larger estimates than cross-sectional designs and field-specific differences remaining significant. Geographic/economic context was not a significant moderator, while trait EI was stronger than ability EI among emotional measures. Claims about incremental variance should be removed unless supported by a separate variance-decomposition analysis.

## 5 Discussion

### 5.1 Key findings

The corrected synthesis of the findings was more restrictive in its conclusions than the initial synthesis, with cognitive predictors showing the most consistency and emotional and behavioral findings showing less consistency and therefore should be kept in mind with caution. The pattern indicates that the most consistent evidence in the studies included is related to academic preparation and cognitive factors. Compared to emotional and behavioral predictors, there is more variability and less clarity in the available quantitative evidence of their role in achievement (Schneider and Preckel, 2017).

Cognitive predictors had the strongest positive association, and emotional and behavioral predictors had weaker and less consistent links. This pattern suggests that cognitive factors are still the most informative single domain, but that behavioral self-regulation and emotional functioning may also provide informative but less stable information (Bücker et al., 2018; Schneider and Preckel, 2017).

There is an additional nuance added by the moderator analyses. Longitudinal studies offer greater effects and do not necessarily yield stability of measurement of these constructs over time, and may be less stable than cross sectional studies because only a single point in time was measured. A greater effect in health and medical programs could be due to higher stakes, increased structure, or greater selection effects that amplify health program performance relationships between preparation and self-regulation (Donker et al., 2014). In particular, among the emotional competencies, the trait EI assessments demonstrated higher correlations with achievement than ability-based assessments, indicating that the student’s perceived emotional competencies and emotional disposition may be more likely to be correlated with the actual learning behavior in the real world than with the abstract EI tasks (Stockinger et al., 2021).

### 5.2 Practical implications

The practical implications should be framed conservatively, with stronger emphasis on cognitive preparation and only tentative support for behavioral and emotional interventions. The findings have implications for institutions, teachers and support services for students. First, there is a need to continue to rely on the basics of cognitive preparation: Admissions criteria and early diagnostic assessment that captures prior knowledge and core skills still provide critical information about students’ academic risk. But the fact that there is extra error variance attributable to emotional and behavioral predictors suggests that using cognitive indicators alone will systematically underestimate a significant portion of the predictors of success or failure. Self-regulation, time management, and motivational–emotional profiles could be incorporated into the early warning system to better identify students with poor study habits and/or emotional stress that may seem “strong” on paper.

Second, the effects are providing clear targets for behavioral and emotional predictors for intervention. Courses specifically designed to teach SRL strategies, time management and active study habits will probably benefit students’ grades, particularly in the first year of university where demands are different. Likewise, interventions designed to decrease maladaptive emotions (such as test anxiety, catastrophizing) and to increase adaptive emotions (such as reappraisal, academic resilience, positive achievement emotions) can be reasonably predicted to help performance. The present findings indicate that academic achievement is influenced by multiple interrelated cognitive, emotional, and behavioral factors. Consequently, interventions are likely to be more effective when they integrate cognitive support (e.g., bridging courses), behavioral skills training (e.g., learning strategy workshops), and psychosocial support (e.g., emotion regulation or stress management programs) rather than focusing on a single domain alone.

Lastly, the moderation throughout the field and design provides a guide to where and how support will be offered. Self-regulation training and managing emotions during embedded training may be particularly beneficial in health and medical programs where stakes and workload are high. Longitudinal studies have indicated that early intervention and continuing supports over time, as opposed to one-off workshops will be most likely to capture the full benefit of these predictors. Instructors can help by structuring courses to incentivize distributed practice, engagement and reflective learning, helping students to make use of their behavioral and emotional strengths instead of depending on ability to take them to the finish (Richardson et al., 2012).

### 5.3 Limitations

There are a number of caveats to temper such conclusions. The overall sample size of studies is large, but the effects are derived from many studies that are cross section in design and therefore limit strong causal inferences. The observed relationships are in line with the notion that cognitive, emotional, and behavioral processes do affect achievement, but there are also reverse causalities and reciprocal relations such as low grades decreasing self-efficacy, and therefore triggering anxiety. The heterogeneity statistics suggest significant differences among studies, which can be attributed to differences in population, discipline, measurement tools and analytical approaches. Subgroup and meta regression analyses accounted for some of this variation, but much of it cannot be accounted for it.

Another issue is the limitations of measurement. Constructs such as self-regulation, emotional intelligence and engagement were measured by a diversity of measures, some more psychometrically sound than others. Social desirability and shared method variance with self-reported GPA could be particularly problematic for self-report measures. As in the case of conceptually related sub scales that are assigned to a broad domain (e.g., as different learning strategies under “behavioral”), nuances are likely to be compressed, and discrete mechanisms may be lost. The small significance values for ability EI may be due to the emotional domain, both because of actual relationships and measurement issues. Despite the publication bias checks, the meta-analysis inevitably required studies to provide enough statistical data to calculate effect sizes, and this may have favored reporting of better studies or more statistically significant studies.

### 5.4 Directions for future research

Longitudinal and experimental designs are needed in future studies to reduce the confounds of temporal ordering and causal pathways among the cognitive, emotional, and behavioral predictors. Studies that examine these constructs repeatedly over transitions (e.g., across secondary school to university, or across years of university) would contribute to understanding the relationship between changes in self-regulation, emotions, and study behavior with changes in achievement. There is a need for more multidimensional, fine-grained assessment of self-regulated learning and emotion processes, including the possibilities for integrating measures of self-report, behavioral logging (e.g., learning management system data), and performance tasks to limit reliance on single method assessments.

The other exciting avenue that could be explored is the testing of integrated interventions targeting multiple domains and the examination of them using sound designs. The scaffolding of prior knowledge and training in planning and monitoring strategies, for instance, in combination with emotion regulation strategies, for example, could be considered as a more realistic test of how these predictors interact in real classrooms. More focus should also be given to moderation by context: field of study, institutional type, cultural context, and digital learning environments may influence the factors that have the greatest impact and at what time. There is a need for more evidence from low income and under-represented disciplines to enhance the generalizability of the current evidence base.

## 6 Conclusion

The findings of the current systematic review and meta-analysis suggest that the cognitive predictors tend to show stronger relationships with academic achievement than do the emotional and behavioral predictors. Although there are some relations between studies, there is considerable between-study variation indicating that the relations may differ across educational settings and populations. The results indicate a moderate pattern that cognitive predictors are the most reliable, and the emotional and behavioral predictors are still tentative in the existing quantitative data set.

## Data Availability

The original contributions presented in the study are included in the article/Supplementary material, further inquiries can be directed to the corresponding authors.
