# Supplementary material for: Meta-analysis of cognitive, emotional, and behavioral predictors of academic achievement in higher education students
Source: Front Psychol. 2026 Aug 6;17:1910328. doi: 10.3389/fpsyg.2026.1910328 (PMC13490195; doi:10.3389/fpsyg.2026.1910328)
Supplement: Supplementary file 1 [file Table_1.DOCX]

# Table S1. Hypothesis Testing Summary

| **H#** | **Hypothesis** | **Evidence** | **Statistical Test** | **Decision** | **Interpretation** | **Support** |
| --- | --- | --- | --- | --- | --- | --- |
| H1 | All three domains show significant associations with academic achievement | Cognitive rˉ=0.197\bar{r}=0.197rˉ=0.197; Emotional rˉ=−0.105\bar{r}=-0.105rˉ=−0.105; Behavioral rˉ=0.002\bar{r}=0.002rˉ=0.002 | Pooled effects in Table 4 | Partially supported | Cognitive was the clearest positive association; emotional was weaker and behavioral was essentially null. | Partial |
| H2a | Behavioral predictors add information beyond cognitive indicators | Behavioral vs cognitive contrast | Table 7 meta-regression, b=−0.190b=-0.190b=−0.190, p<.001p<.001p<.001 | Supported as a domain difference | Behavioral differed from cognitive, but no variance-explained claim should be made here. | Partial |
| H2b | Emotional predictors add information beyond cognitive indicators | Emotional vs cognitive contrast | Table 7 meta-regression, b=−0.474b=-0.474b=−0.474, p<.001p<.001p<.001 | Supported as a domain difference | Emotional differed from cognitive, but no variance-explained claim should be made here. | Partial |
| H3 | Cognitive domain shows the strongest pooled effect | Cognitive vs emotional vs behavioral | Z=2.81Z=2.81Z=2.81, p=.005p=.005p=.005 for cognitive vs emotional | Partially supported | Cognitive was stronger than emotional; cognitive vs behavioral should be described cautiously. | Partial |
| H4 | Study design moderates effects | Longitudinal/SEM vs cross-sectional | Table 6 and Table 7 | Supported | Longitudinal/SEM studies yielded larger estimates. | Full |
| H5 | LMIC context moderates effects | LMIC vs non-LMIC | p=.200p=.200p=.200 | Not supported | Geographic/economic context was not a significant moderator. | Not supported |
| H6 | Field of study moderates effects | Across fields | QB=9.82QB=9.82QB=9.82, p<.001p<.001p<.001 | Supported | Field of study significantly moderated effects. | Full |
| H7 | EI measurement type moderates emotional effects | Trait vs ability EI | QB=5.91QB=5.91QB=5.91, p=.015p=.015p=.015 | Supported | Trait EI showed stronger effects than ability EI. | Full |
